# MicroRNA-1246 by Targeting AXIN2 and GSK-3β Overcomes Drug Resistance and Induces Apoptosis in Chemo-resistant Leukemia Cells

**DOI:** 10.7150/jca.58522

**Published:** 2021-05-13

**Authors:** Bei Xie, Linjing Li, Zhewen Zhang, Lei Zhao, Juan Cheng, Cunmin Zhou, Jie Cheng, Jing Yan, Jing Chen, Juan Yi, Bei Wang, Suya Jin, Hulai Wei

**Affiliations:** 1 Department of Medical Laboratory Animal Science, School of Basic Medical Sciences, Lanzhou University, Lanzhou, Gansu, 730000.; 2 The Second Hospital of Lanzhou University, Lanzhou, Gansu, 730000.; 3 Shaanxi Meili Omni‑Honesty Animal Health Co., Ltd., Xi'an, Shaanxi, 710000.; 4 The First Hospital of Lanzhou University, Lanzhou, Gansu, 730000.

**Keywords:** leukemia cells, miR-1246, chemo-resistance, AXIN2, GSK-3β, Wnt/β-catenin pathway.

## Abstract

**Background and objective:** Chemotherapy plays an important role in the treatment of leukemia. Multidrug resistance (MDR) induced by chemotherapy always leads to treatment failure and disease recurrence. MicroRNAs (miRNAs) have been verified as crucial components in carcinogenesis, including chemo-resistance of tumor cells, which has not been fully understood. In this study, we aimed to identify the potential candidate miRNA, miR-1246, and reveal its regulatory role in chemo-resistance of leukemia cells.

**Methods:** Candidate miRNAs were selected by microarray analysis, screened by bioinformatics tools and verified by reverse transcription-quantitative polymerase chain reaction (RT-qPCR). Chemo-resistant phenotypes, including cell viability, apoptosis, adriamycin (ADM) efflux and *in vivo* oncogenicity of leukemia cells following transfected with miR-1246 mimics or inhibitor were checked with or without ADM treatment to make clear the relationship between miR-1246 and chemo-resistance. RT-qPCR, western blot and dual luciferase reporter assay were performed to measure the expression of related genes and address the potential regulatory mechanism of miR-1246 in chemo-resistance.

**Results:** The expression of miR-1246 was significantly higher in chemo-resistant leukemia K562/ADM cells, HL-60/RS cells and recurrent primary leukemia cells. Loss of miR-1246 inhibited proliferation, induced apoptosis, altered cell cycle distribution, inhibited ADM efflux in chemo-resistant leukemia cells, while overexpression of miR-1246 showed the opposite role in chemo-sensitive leukemia cells. Both bioinformatics prediction and luciferase assay indicated that AXIN2 and glycogen synthase kinase 3 beta (GSK-3β) were the direct targets of miR-1246 in leukemia cells. Inhibition of miR-1246 could up-regulate AXIN2 and GSK-3β and inactivate Wnt/β-catenin pathway, accompanied with inhibiting the expression of β-catenin and further influencing the expression of P-glycoprotein (P-gp) in the chemo-resistant leukemia cells.

**Conclusions:** Chemo-resistant ability of MDR leukemia cells is attenuated by loss of miR-1246 via negatively regulating AXIN2 and GSK-3β to inactivate Wnt/β-catenin pathway and suppress P-gp expression, these mean that targeting miR-1246-AXIN2/GSK-3β-Wnt/β-catenin axis may be beneficial to overcome the chemo-resistance in relapse and refractory leukemia patients.

## Introduction

Leukemia, ranking the first place in young people's malignant diseases, is the malignant tumor of hematopoietic system 1. At present, chemotherapy is still an important treatment method for leukemia. But with the use of chemotherapy drugs, leukemia cells gradually develop resistance resulting in chemotherapy failure 2. The inherited (natural) or acquired drug resistance, especially multidrug resistance (MDR) is considered to be the major cause for chemotherapy failure 3. Despite of distinct mechanisms, MDR is usually the cooperative effect of a combination of MDR mechanisms such as blocking apoptosis and increasing drug efflux 4. Among drug efflux transporters, the most extensively studied is adenosine triphosphate-binding cassette (ABC) transporters. The first discovery and well characterized is P-glycoprotein (P-gp/MDR1/ABCB1) 5. P-gp is currently considered to be one of main hindrances in the anticancer therapy and functions as a drug efflux pump and causes maintenances of tolerable intracellular levels of the cytotoxic drugs 4, 6, 7. Recently, microRNAs (miRNAs) have attracted much more attention in a variety of cancers, including leukemia.

miRNAs are small non-coding RNAs with a length of 18-25 nucleotides, involved in regulating gene expression posttranscriptionally 8, 9. It has been estimated that miRNAs could regulate ~60% of human genes and are implicated in various biological processes such as cell cycle control, apoptosis, metabolism, development, differentiation 9. Mechanistically, miRNAs can act as both tumor suppressors and oncogenes by silencing different gene expression in various types of cancer 10. Emerging studies prove that miRNAs are involved in chemo-resistance via multiple transduction pathways 10. The mutation and abnormal expression of miRNAs can alter the post transcriptional regulation of target genes, resulting in abnormal expression of target genes, and ultimately alter the drug sensitivity of tumor cells through cell signaling pathways. The target genes related to drug sensitivity mainly include the genes related to drug transport, drug target, drug detoxification, apoptosis, cell repair and cell cycle regulation 11-21, among which apoptosis and drug transport related genes are the most studied. Evidences have proved that some kinds of miRNAs are ectopic expressed, resulting in affecting the expression of drug resistance genes, including MDR1 22, 23. miR-122 could directly target and suppress Wnt/β-catenin pathway, while β-catenin bound with MDR1 promoter and activated its transcription, thus inducing cell apoptosis 24. miR-381 could overcome cisplatin (DDP) resistance of breast cancer by directly targeting MDR1 and inducing cell apoptosis 25. Overexpression of miR-506 could enhance oxaliplatin sensitivity by inhibiting MDR1/P-gp expression via down-regulating the Wnt/β-catenin pathway and increasing cell apoptosis 26. Ectopic expression of miR-298 could directly bind to the 3' untranslated region (3' UTR) of P-gp and down-regulate its expression 27.

In this study, we had screened an abnormal expressed and drug resistance-related miRNA, miR-1246, by microarray and sought to investigate the possible molecular mechanisms underlying the role of this miRNA in regulating the chemo-resistance in leukemia cells.

## Materials and Methods

### Materials

miR-1246 mimics, NC mimics, miR-1246 inhibitor and NC inhibitor, Hairpin-it™ miRNAs qPCR Quantitation Kit (cat. no. E01006) and the specific primer settings for miR-1246 and U6 were purchased from Shanghai GenePharma Co., Ltd (Shanghai, China). SYBR Premix EX Taq™ and PrimeScript™ RT kits were obtained from Takara Bio, Inc (Otsu, Japan). Annexin V/dead cell apoptosis kit (cat. no. V13241) was procured from Invitrogen, Thermo Fisher Scientific, Inc (Waltham, MA, USA). Dual-Glo® Luciferase Assay System (cat. No. E2920) and pRL-TK Renilla Luciferase Control Reporter Vectors (cat. no. E2241) were obtained from Promega corporation (Madison, WI, USA). pMIR-REPORT Luciferase vector (cat. no. VT1399) was purchased from Ambion Corporation (Austin, TX, USA). 2×Hieff® Robust PCR master Mix (with Dye) (cat. no. 10106ES03) was purchased from Yeasen Biotech Co., Ltd (Shanghai, China). FastDigest SacI (cat. no. FD1133) and FastDigest MluI (cat. no. FD0564) were procured from Thermo Fisher Scientific, Inc (Waltham, MA, USA). Antibodies specific for AXIN2 (cat. no. ab109307; Abcam), glycogen synthase kinase 3 beta (GSK-3β) (cat. no. ab32391; Abcam), β-catenin (cat. no. sc-7963; Santa Cruz Biotechnology, Santa Cruz, CA, USA), Wnt2 (cat. no. ab109222; Abcam), adenomatous polyposis coli (APC) (cat. no. sc-896; Santa Cruz Biotechnology, Santa Cruz, CA, USA), c-Myc (cat. no. ab32072; Abcam), P-gp (cat. no. #13342; Cell Signaling technology) and β-actin (cat. no. TA-09; Zhongshan Jinqiao Bio-Technology Co., Ltd.) were used in the present study.

### Ethics statement

All the experiments were undertaken following the provisions of the Declaration of Helsinki. This study was approved by the Ethics Committee of the First Hospital of Lanzhou University (Gansu, China). All patients provided written informed consents for the collection of samples and subsequent analysis. All animal studies were in accordance with the animal protocol approved by the Laboratory Animal Science and Technology Management Committee of Lanzhou University School of Basic Medical Sciences (Gansu, China).

### Blood samples and RNA extraction

A total of 14 leukemia patients (6 male and 8 females aged 15-67 years) were recruited from the Hematology Department of the First Hospital of Lanzhou University between March, 2017 and March, 2020. These patients were definitely diagnosed as acute myeloid leukemia (AML) and received standard chemotherapy, and all the patients had acquired partial or complete remission. Subsequently, the leukemia patients were relapsing and became entirely resistant to the chemotherapy. The blood samples of the 14 patients before chemotherapy and after recurrence were collected, and the peripheral blood mononuclear cells (primary leukemia cells) were separated using cell density gradient medium (Solarbio, cat. no. P8610, Beijing, China), then total RNA of the mononuclear cells was prepared using the TRIzol reagent (Invitrogen Life Technologies, Carlsbad, CA, USA) and then subjected to reverse transcription-quantitative polymerase chain reaction (RT-qPCR) assay.

### Cell culture and incubation

Human K562, HL-60 cells and drug-resistant K562/ADM cells were purchased from American Type Culture Collection (ATCC; Manassa, VA, USA). HL-60/RS cell subline was stable adriamycin (ADM)-resistant leukemia subline generated from HL-60 cells by ADM-selection in our laboratory, which possessed the typical features of multidrug resistance 28. All the cells were maintained in RPMI-1640 (Gibco; Thermo Fisher Scientific, Inc.) supplemented with 10% fetal bovine serum (Hyclone; GE Healthcare Life Sciences, Logan, UT, USA) and cultivated at 37˚C in a humidified incubator, containing 5% CO_2_. In the latter experiments, the cells were divided into the groups as chemo-sensitive groups transfected with NC mimics, NC mimics + ADM, miR-1246 mimics and miR-1246 mimics + ADM, respectively or chemo-resistant groups transfected with NC inhibitor, NC inhibitor + ADM, miR-1246 inhibitor and miR-1246 inhibitor + ADM, respectively.

### miRNA microarray and analysis

Total RNA was extracted from K562 and K562/ADM cells and quantified by microfluidic analysis. miRNA microarray expression profiling was performed by Exiqon® (Vedbaek, Denmark) as a commercial service using their seventh generation miRCURY™ LNA Array microarray platform. miRNA microarray data was analyzed by subtracting the background and then normalizing the signals with a LOWESS filter (Locally-weighted Regression). Differentially expressed miRNAs were defined when the *p* value was less than 0.05, and most notable differentially expressed miRNAs were defined when the *p* value was less than 0.01. Those who were most notable differentially expressed miRNAs related to multidrug resistant characteristic were further investigated.

### RNA isolation and RT-qPCR assay

RNA isolation was performed as previously described 29. Total RNA from the cells was isolated using TRIzol® reagent (Invitrogen; Thermo Fisher Scientific, Inc.). For miRNAs levels, detection and quantification of miRNAs from total RNA samples were done using the Hairpin-it™ miRNAs qPCR Quantitation Kit purchased from Shanghai GenePharma Co., Ltd (Shanghai, China) according to the manufacture's protocol. RT-qPCR was performed using a Rotor-Gene 3000 quantitative PCR amplifier (Corbett Life Science; Qiagen, Inc.). The following primers were purchased from Shanghai GenePharma Co., Ltd (Shanghai, China): miR-1246 mimics, AAUGGAUUUUUGGAGCAGG, NC mimics, UUCUCCGAACGUGUCACGUTT; and miR-1246 inhibitor, CCUGCUCCAAAAAUCCAUU, NC inhibitor, CAGUACUUUUGUGUAGUACAA. The miRNA concentration was normalized to the endogenous control U6.

For gene mRNAs detection, total RNA was reverse transcribed to cDNA using a PrimeScript RT reagent kit purchased from Takara Bio, Inc. (Otsu, Japan) according to the manufacture's protocol. qPCR was performed using a SYBR Premix Ex Taq II kit. qPCR was conducted as follows: 10 sec at 95˚C, followed by 35 cycles of denaturation at 95˚C for 5 sec and annealing at 60˚C for 30 sec. β-actin was used as the internal control. The relative expression levels of the genes were determined by the 2^-ΔΔCq^ method.

### Transfection

For the miR-1246 functional analysis, miR-1246 mimics and NC mimics (GenePharma, Shanghai, China) were transfected into chemo-sensitive K562 and HL-60 cells using Lipofectamine 2000 (Invitrogen; Thermo Fisher Scientific, Inc.) according to the manufacturer's protocols. In the same way, chemo-resistant K562/ADM and HL-60/RS cells were transfected with miR-1246 inhibitor or NC inhibitor (GenePharma, Shanghai, China). 48 h after transfection, they were collected and subjected for further analysis.

### Cell viability assay

A cell viability assay was performed as previously described 30. Briefly, cells in exponential growth phase were plated at a final concentration of 1
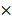
10^4^ cells/well into 96-well culture plates. The viability of the cells was determined by MTT assay 24, 48 and 72 h following seeding. A total of 10 μL MTT solution was then added to each well, followed by incubation for 4 h at 37˚C. 10% SDS was added to each of the wells, and incubated overnight at 37˚C to dissolve the formazan crystals. Finally, the absorbance values of each well were measured at 570 nm, and the readings were quantified using a Powerwave X plate reader (Bio-Tek Instruments, Inc.). Cell proliferation inhibitory rates were calculated.

### Morphological characteristics

The morphological characteristics of the cells were observed from the microscopic and ultrastructural levels. The general process was as follows, after the cells were seeded, they were transfected with NC inhibitor or miR-1246 inhibitor. Subsequently, they were treated with or without ADM and observed under an inverted microscope (IX81; Olympus Corporation).

After the cells were collected, they were sequentially fixed with 2% glutaraldehyde and 1% OsO_4_ for ultrastructural observation as previously performed 29.Following dehydration, embedded and staining, they were observed under a JEM 1230 transmission electron microscope (JEOL, Ltd.). Images were acquired digitally from a randomly selected pool of 10-15 fields for each condition at 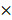
10,000 magnification.

### Flow cytometry assay

The apoptotic analysis was determined using an Annexin V/dead cell apoptosis kit (cat. no. V13241; Invitrogen; Thermo Fisher Scientific, Inc.). Samples were gently suspended in 100 μL binding buffer containing 5 μL of Annexin V-FITC and 5 μL propidium iodide (PI), and further incubated for 15 min in the dark at room temperature. Finally, cells were suspended in 500 μL binding buffer and detected by flow cytometry using FACSVerse (BD Biosciences, San Jose, CA, USA). The apoptotic rate was determined for each condition as follows: Apoptotic rate = (early apoptotic rate + late apoptotic rate) × 100%.

Cells were washed with ice-cold PBS and fixed in cold 70% ethanol overnight. Prior to analysis, cells were washed with PBS and incubated with PI for 30 min at room temperature in the dark. The DNA contents were analyzed with Multicycle for Windows using a FACSVerse flow cytometer (BD Biosciences, San Jose, CA, USA).

### ADM efflux assay

This experiment was determined by flow cytometry as previously described 28. In order to detect the ability of efflux, cells were incubated with 30 mg/L ADM at 37˚C for 30 min. Following two washes with PBS, 1
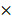
10^6^ cells were incubated at 37˚C for another further 60 min and then detected for their efflux ability immediately by using a FACSVerse flow cytometer (BD Biosciences, San Jose, CA, USA).

### miR-1246 target genes selection

miR-1246 target genes were selected using several target prediction programs, including miRDB, miRWalk, PICTAR4, Targetscan, DIANA and miRanda. All the miR-1246 target genes were screened further by GenBank and these related to multidrug resistance were selected. AXIN2 and GSK-3β, which were the most relevant to miR-1246 and MDR, were selected for further investigation.

### Western blot assay

Western blot analysis was performed as previously described 29. After the cells were lysed with RIPA lysis buffer (Beijing Solarbio Science & Technology Co. Ltd.), proteins were collected and their concentrations were determined using the BCA method. The proteins (40 μg) were separated on 10% sodium dodecyl sulfate-polyacrylamide gel electrophoresis (SDS-PAGE) gels and were subsequently transferred onto polyvinylidene membranes (EMD Millipore, Bedford, MA, USA). Following blocking in 0.1% TBS-Tween 20 containing 5% nonfat milk at room temperature for 1 h, the membranes were incubated overnight at 4˚C with primary antibodies AXIN2 (1:2000; cat. no. ab109307; Abcam), with GSK-3β (1:2000; cat. no. ab32391; Abcam), or with β-actin (1:1,000; cat. no. TA-09; Zhongshan Jinqiao Bio-Technology Co., Ltd.) as the control. The following morning, the membranes were incubated at room temperature for 1 h with horseradish peroxidase conjugated goat anti-mouse (cat. no. SA00001-1) or goat anti-rabbit (cat. no. SA00001-2) secondary antibodies (1:5,000; Proteintech Group, Inc.) and subsequently developed using an Amersham Enhanced Chemiluminescence Western blot detection system (GE Healthcare Life Sciences) according to the manufacturer's protocols.

### Dual luciferase reporter assay

Luciferase assay was performed following the previous method 31. Both 3' UTR of AXIN2 (nt 304-321) and 3' UTR of GSK-3β (nt 497-517), containing miR-1246 binding sites, were amplified, cloned and inserted into a pMIR-REPORT luciferase vector in sense or anti-sense directions using MluI and SacI at the restriction enzyme cutting sites. 293T cells were seeded in 96 well plates 1 day before transfection. Then, 293T cells were co-transfected with AXIN2 or GSK-3β 3'-UTR pMIR-REPORT luciferase vector, pRL-TK reporter vector and miR-1246 mimics or NC mimics using Lipofectamine 2000. 48 h after transfection, firefly and renilla luciferase activities were measured using the Dual-Glo® Luciferase Assay System on a FlexStation 3 Multi-Mode Microplate Reader (Molecular Devices, CA, USA) according to the manufacturer's instructions. Relative luciferase activity was normalized with renilla luciferase activity.

### *In vivo* xenograft mouse model

Sixty male BALB/c nude mice (specific pathogen-free grade, 5 weeks old, 18-22 g) were purchased from Vital River Laboratories (Beijing, China). The nude mice were housed in the barrier system facilities of Department of Medical Laboratory Animal Sciences, School of Basic Medical Sciences, Lanzhou University (Gansu, China). All the animal procedures were ethically approved by the Laboratory Animal Science and Technology Management Committee of the Lanzhou University School of Basic Medical Sciences, and were conducted in accordance with the Guide for Care and Use of Laboratory Animals 29, 31. Mice were randomly divided into six groups (Mock, NC inhibitor, miR-1246 inhibitor, Mock + ADM, NC inhibitor + ADM, miR-1246 inhibitor + ADM) and injected with transfected K562/ADM-GFP cells (2×10^6^ cells) through subcutaneous axillary injection and treated with 7 mg/kg ADM through intraperitoneal injection every week. Every 7 days, mice in each group were analyzed using a Photon Imager for GFP activity using the Lumina II Living Image 4.0 software (PerkinElmer, Inc.). After 30 d, the mice were sacrificed and the tumors were harvested, measured and weighed.

### Statistical analyses

The data were presented as the mean ± standard deviation from at least triplicate experiments performed three times. Statistical analyses were performed using SPSS 16.0 software (SPSS, Inc., Chicago, IL, USA). The difference between two groups was analyzed using Student's t-test. *P* < 0.05 was considered to indicate a statistically significant differences.

## Results

### miR-1246 was preferentially overexpressed in chemo-resistant leukemia cell lines and recurrent primary leukemia cells

**Figure 1:**
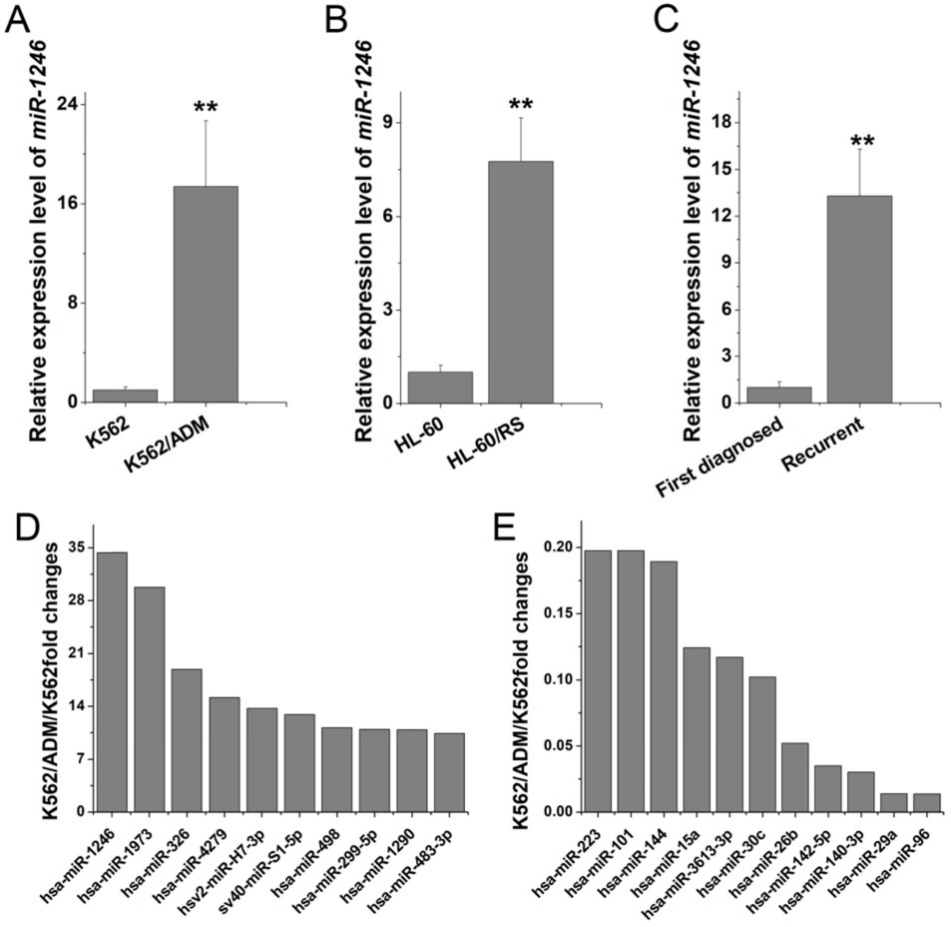
miR-1246 was highly expressed in chemo-resistant leukemia cells and recurrent primary leukemia cells. A and B, miR-1246 was detected in K562, K562/ADM cells and HL-60, HL-60/RS cells using RT-qPCR. C, RT-qPCR assay was used to testify the expression difference of miR-1246 between the first diagnostic primary leukemia cells and recurrent primary leukemia cells in patients (** P < 0.01, comparing to their parental cells or the first diagnostic samples). D and E, The expression levels of the candidate miRNAs in K562/ADM cells compared to their parental cells.

To explore which miRNAs may take part in and play a vital role in chemo-resistant regulation in leukemia cells, miRNA microarray studies were performed to identify the candidate miRNAs. Comparing to K562 cells, 10 miRNAs were elevated (fold change > 10-fold) and 11 miRNAs were decreased (fold change < 0.2-fold) in K562/ADM cells (Table 1). Among them, miR-1246 showed the highest alteration (34.36-fold increase in K562/ADM cells). RT-qPCR confirmed that the expression level of miR-1246 was significantly higher in multidrug resistant K562/ADM (17.41-fold change) and HL-60/RS (7.76-fold change) cells (Figure 1A and B) than in their parental sensitive cells. Additionally, overexpression of miR-1246 was also found in recurrent primary leukemia cells than in the first diagnostic primary leukemia cells in patients (Figure 1C). All these results indicated that miR-1246 was overexpressed in chemo-resistant leukemia cells and recurrent primary leukemia cells, which suggested that miR-1246 might be an onco-miR related to chemo-resistance in leukemia. Hence, miR-1246 was selected to investigate its association with the chemo-resistance of leukemia cells to ADM and disclose its potential mechanism.

### Inhibition of miR-1246 increased chemo-sensitivity of drug-resistant leukemia cells

**Figure 2:**
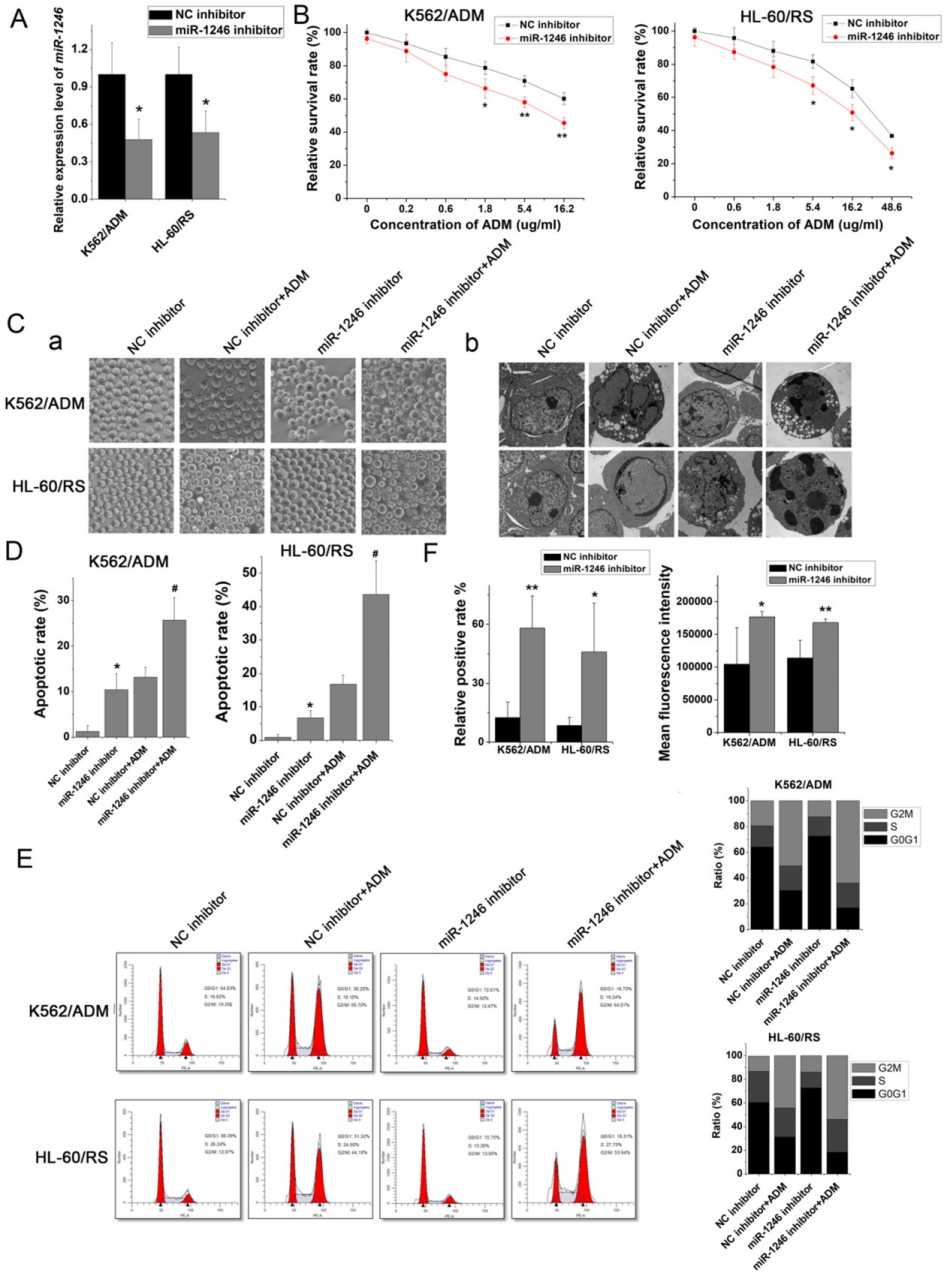
Loss of miR-1246 in drug-resistant leukemia cells could improve their drug sensitivity and induce cell apoptosis. A, The expression of miR-1246 was inhibited in chemo-resistant leukemia cells (* P < 0.05, comparing to their negative control, respectively). B, The cell survival and proliferation rate was measured using MTT assay after the miR-1246 was repressed in K562/ADM and HL-60/RS cells (* *P* < 0.05, ** *P* < 0.01, comparing to the NC inhibitor transfected cells treated with the same concentration of ADM, respectively). C, Morphological alterations of K562/ADM and HL-60/RS cells transfected with NC inhibitor or miR-1246 inhibitor and treated with or without ADM. (a) Morphological changes detected by phase-contrast microscopy (×200). (b) Ultrastructural changes of cells dectected by transmission electron microscopy (×10,000). D, The cell apoptotic rate was examined using flow cytometry staining with Annexin V-FITC and PI (* P < 0.05, comparing to their negative control. # *P* < 0.05, comparing to their negative control treated with the same concentration of ADM). E, After the cells were fixed and stained, flow cytometry was used to check the cell cycle distribution. F, Relative positive rate and mean fluorescence intensity were analysed to investigate the ADM efflux ability of cells using flow cytometry. (* *P* < 0.05, ** *P* < 0.01, comparing to their negative control, respectively).

To investigate and illuminate the relationship between miR-1246 and chemo-resistance in leukemia cells, the cell viability assay, apoptotic analyses, cell cycle distribution analyses and ADM efflux assay were performed to check the sensitivity of drug-resistant leukemia cells to ADM after miR-1246 was inhibited. Comparing to the NC inhibitor transfected K562/ADM cells and HL-60/RS cells, the miR-1246 expression was successfully repressed in K562/ADM cells and HL-60/RS cells transfected with miR-1246 inhibitor (Figure 2A). The results of cell viability assay showed that ADM treatment could repress the cell survival rate and inhibit the cell proliferation ability. After miR-1246 was restrained, the cell survival and proliferation rate was even lower as shown in Figure 2B. The alteration of morphology was demonstrated by phase-contrast microscopy and transmission electron microscopy (Figure 2C). Suppression of miR-1246 could significantly increase the sensitivity of chemo-resistant leukemia cells to ADM and make the apoptosis more obvious. After Annexin V-FITC and PI staining, the data checked by flow cytometry showed that loss of miR-1246 promoted cell apoptosis, and this was statistically significant (Figure 2D). Similar to previous cell cycle results at the small extracellular vesicle level 32, lack of miR‐1246 in chemo-resistant leukemia cells repressed the advancement of cell cycle by halting cells at the G0/G1 phase. While ADM treatment led to cell cycle arrest and the accumulation of cells at the G2/M phase (Figure 2E). Furthermore, miR-1246-knockdown could significantly reduce the ADM efflux ability of chemo-resistant K562/ADM and HL-60/RS cells (Figure 2F). All these data suggested that miR-1246 inhibition could reverse the multidrug resistant ability and increase their chemo-sensitivity of K562/ADM and HL-60/RS cells to ADM by inhibiting the cell proliferation ability, halting the cell cycle distribution and inducing cell apoptosis.

### Overexpression of miR-1246 generated chemo-resistance in drug-sensitive leukemia cells

**Figure 3:**
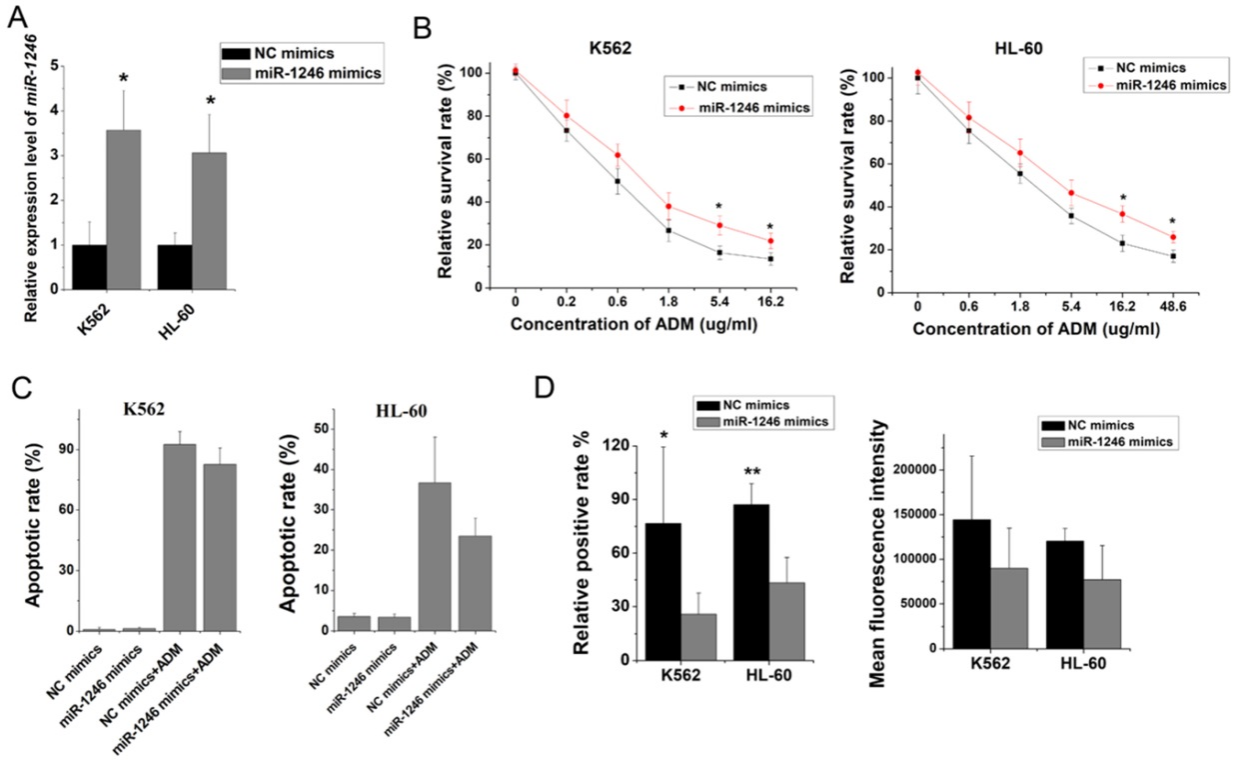
Overexpression of miR-1246 could improve the chemo-resistant ability of drug-sensitive leukemia cells. A, miR-1246 was checked using RT-qPCR after K562 and HL-60 cells transfected with miR-1246 mimics or NC mimics (* *P* < 0.05, comparing to their negative control, respectively). B, MTT assay was used to examine the cell survival and proliferation rate of miR-1246 mimics or NC mimics transfected drug-sensitive cells treated with different concentration of ADM (* P < 0.05, ** P < 0.01, comparing to their negative control treated with the same concentration of ADM, respectively). C, After the cells were stained with Annexin V-FITC and PI, flow cytometry was used to detect their cell apoptotic rate (* P < 0.05, comparing to their negative control. #* P* < 0.05, comparing to their negative control treated with the same concentration of ADM). D, ADM efflux ability was measured using flow cytometry after the cells were incubated with ADM (* P < 0.05, ** P < 0.01, comparing to their negative control, respectively).

To further clarify the role of miR-1246 in chemo-resistance, overexpression of miR-1246 in chemo-sensitive leukemia cells was also performed. As shown in Figure 3A, the expression of miR-1246 was significantly increased after K562 and HL-60 cells were transfected with miR-1246 mimics comparing to NC mimics transfected groups. After the expression of miR-1246 was enhanced, the ability of cell to tolerate ADM was significantly strengthened (Figure 3B). The results of flow cytometry displayed that the increased expression of miR-1246 could abate the ability of ADM to induce apoptosis in chemo-sensitive leukemia cells (Figure 3C). The ADM efflux ability checked by flow cytometry showed that the increase of miR-1246 could heighten the capacity of drug-sensitive leukemia cells to pump ADM and lessen drug damage to them (Figure 3D). All these results proved that overexpression of miR-1246 generated drug-resistance in chemo-sensitive leukemia cells.

### Loss of miR-1246 decreased* in vivo* chemotherapy resistance of drug-resistant leukemia cells

**Figure 4:**
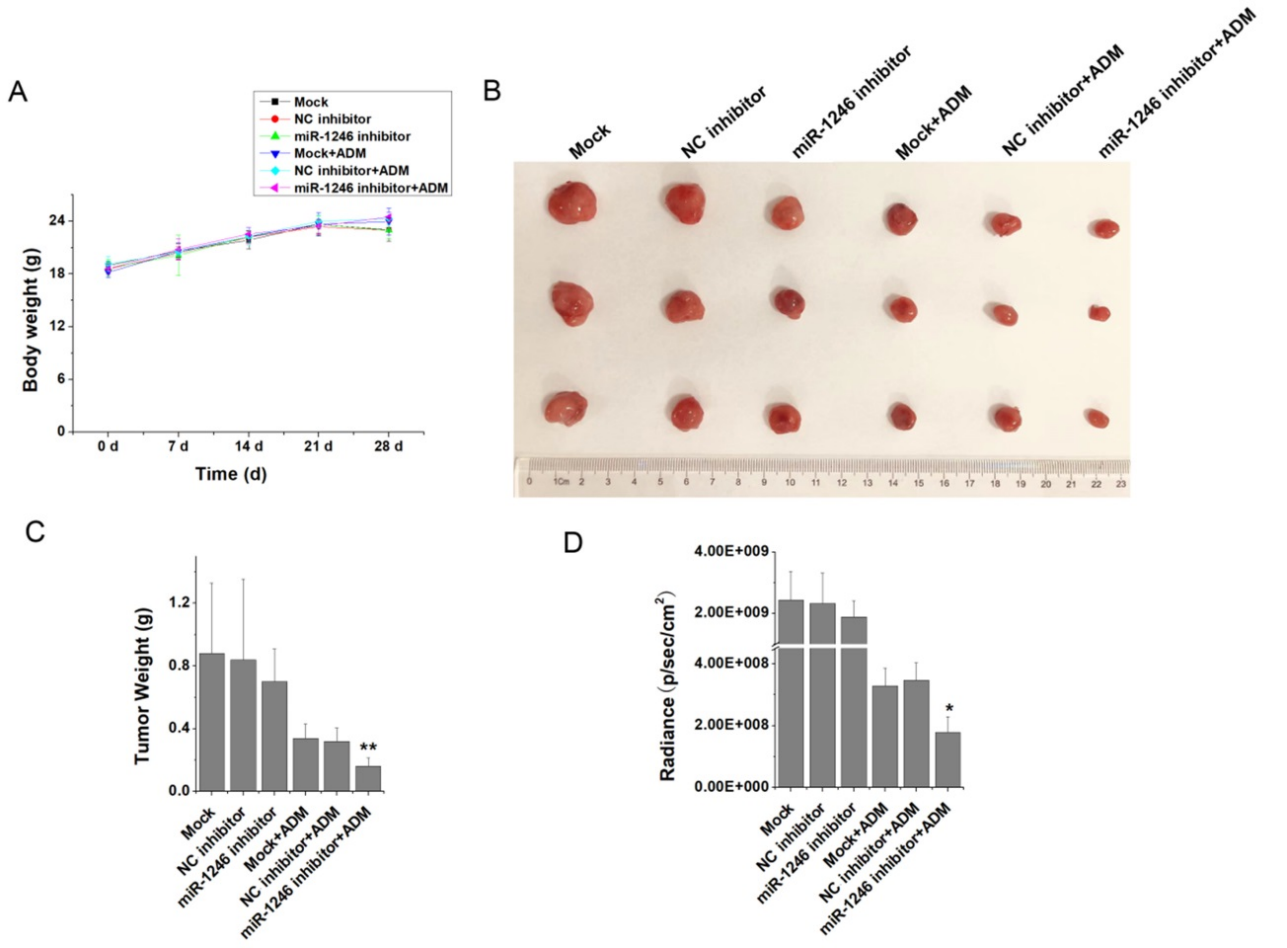
Animal studies were performed and the results were analyzed. A, The weight of mice in all groups were recorded and compared. B and C, The tumors dissected from all groups were photographed and the statistics results of their weights were analyzed. D, The difference of radiance recorded from the tumor of different groups' mice.

To verify the relationship between miR-1246 and chemo-resistance in leukemia cells, *in vivo* animal experiment was performed. K562/ADM-GFP cells transfected with miR-1246 inhibitor or scramble/negative control were injected into nude mice to generate xenograft model. Animal experimental results showed that the weight differences of the BALB/c nude mice in each group were minor and not significant (Figure 4A). At the end of the experiments, all the mice were killed after the last recording from the Lumina II Living Image 4.0 software and the tumor tissues were removed. As shown in Figure 4B, the tumor volume of miR-1246 inhibitor group was smaller than that of the mock or control group. Mice treated with ADM exhibited even smaller tumor volume. In particular, mice of miR-1246 inhibitor group treated with ADM had the minimum tumor volume. Consistent with this, the weight of tumors in mock and NC inhibitor group was similar, while the weight of tumors dissected from the mice of miR-1246 inhibitor transfected group was lower than that of mock or NC inhibitor transfected group, and the difference was not remarkable. The similar manifestations were observed in these three groups treated with ADM. But the difference was significant between miR-1246 inhibitor + ADM group and NC inhibitor + ADM or mock + ADM group (Figure 4C). The same results could conclude in the radiance of GFP activity from these six groups (Figure 4D). *In vivo* results confirmed that suppression of miR-1246 reduced chemotherapy resistant ability of drug-resistant leukemia cells.

### miR-1246 influenced chemo-sensitivity of leukemia cells by targeting AXIN2 and GSK-3β in Wnt/β-catenin pathway and further regulating P-gp expression

**Figure 5:**
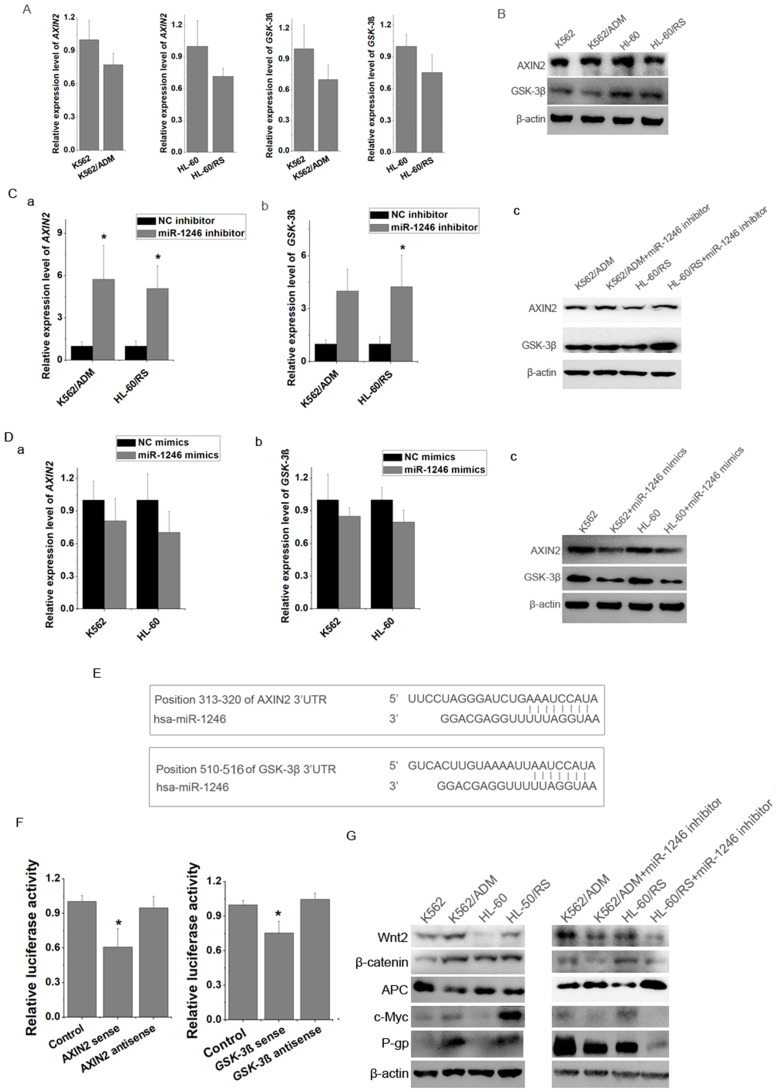
miR-1246 regulated drug-sensitivity of leukemia cells by targeting AXIN2 and GSK-3β and further influencing P-gp activity. A, The mRNA expression level of AXIN2 and GSK-3β in K562/ADM and HL-60/RS cells was analyzed comparing to their chemo-sensitive parental cells (a, b, c, d). B, The protein immunoblotting was used to test the expression of AXIN2 and GSK-3β in K562, K562/ADM and HL-60, HL-60/RS cells. C, Comparing to NC inhibitor transfected chemo-resistant leukemia cells, mRNA (a and b) and protein (c) level of AXIN2 and GSK-3β in miR-1246 inhibitor transfected chemo-resistant leukemia cells were analysed (* *P* < 0.05, comparing to their negative control, respectively). D AXIN2 and GSK-3β were detected using RT-qPCR and western blot in miR-1246 mimics and NC mimics transfected chemo-sensitive leukemia cells. E, Position 313-320 of AXIN2 3'UTR and position 510-516 of GSK-3β 3'UTR were identified as potential targets of miR-1246. F, Dual luciferase reporter assay was performed to verify the interaction between miR-1246 and AXIN2 or miR-1246 and GSK-3β. pRL-TK renilla luciferase plasmid was co-transfected for normalization (* *P* < 0.05, comparing to their negative control, respectively). E, Protein immunoblotting assay was used to check the differences of multidrug resistant protein (P-gp) and key factors in Wnt/β-catenin pathway and its downstream factors with or without miR-1246 inhibitor transfection.

By using miRNA target prediction programs, we found several potential target genes of miR-1246, such as FAM53C, CREBL2, CCNG2, CADM1, AXIN2, GSK-3β, etc. Results showed that the mRNA and proteins levels of AXIN2 and GSK-3β in K562 and HL-60 cells were higher than those in K562/ADM and HL-60/RS cells (Figure 5A and B). Loss of miR-1246 would raise the expression of AXIN2 and GSK-3β in K562/ADM and HL-60/RS cells (Figure 5C). On the contrary, overexpression of miR-1246 could inhibit the AXIN2 and GSK-3β levels in K562 and HL-60 cells (Figure 5D). Bioinformatics tools indicated that one fragment at the 3'UTR of AXIN2 (313-320) and one fragment at the 3'UTR of GSK-3β (510-516) had putative seed-matching sites with miR-1246 (Figure 5E). As shown in Figure 5F, compared with miRNA negative control and anti-sense orientation of fragments transfection, luciferase activity was significantly decreased in the cells transfected with miR-1246 and 3'UTR fragments in the sense orientation, which suggested that miR-1246 could inhibit the expression of AXIN2 and GSK-3β by directly binding to the 3'UTR seed-matching sites.

AXIN2 and GSK-3β are key members of Wnt/β-catenin pathway. Repression of miR-1246 also influenced the expression levels of major components of Wnt/β-catenin pathway and its downstream factors. Loss of miR-1246 could decrease the expression of β-catenin, Wnt2, c-Myc and increase the level of APC (Figure 5G). Previous reports showed that β-catenin could bind with MDR1 promoter and activate its transcription 24. Our further results showed that inhibition of miR-1246 could suppress the activity of P-gp in K562/ADM and HL-60/RS cells (Figure 5G). Based on these, we inferred that miR-1246 regulated the activity of Wnt/β-catenin pathway by targeting AXIN2 and GSK-3β and lastly took part in ADM resistance regulation of leukemia cells.

## Discussion

To date, chemotherapy remains the initial treatment method for patients with leukemia. However, during treatment process, cancers could get intrinsically resistance or acquired resistance to therapies, which would render cancer therapy less effective and finally lead to death. The acquisition of multidrug resistance associated with chemotherapeutic agents has seriously affected the quality of life and survival rate of patients. Multidrug resistance becomes one major challenge responsible for the cancer treatment failure 33. Great progresses have been made in “mechanisms-based” anti-cancer therapy. To combat the multidrug resistance, substantial efforts have been devoted to elucidate the mechanisms underlying the MDR. Growing evidences have confirmed that miRNAs serve as oncogenes or tumor suppressor genes, and also play an important role in carcinogenesis and tumor progression, including drug resistance 3. In the current study, we found that several miRNAs were differential expressed in chemo-resistant leukemia cells and their parental chemo-sensitive cells using miRNA microarray analyses (Table 1). Among them, miR-1246 displayed the most obvious difference. Evidences have suggested that miR-1246 not only can act as an oncogene but also function as a tumor suppressor in different cancer types 34.

Our further verification results proved that miR-1246 was preferentially higher in chemo-resistant leukemia cells than in their parental leukemia cells. Stronger evidence from our clinical samples suggested that miR-1246 was statistically overexpressed in recurrent primary leukemia cells than in the same patient's first diagnostic primary leukemia cells (Figure 1). All these testimonies indicated that miR-1246 might act as oncogene and was differentially expressed due to chemo-sensitive differences in leukemia cells and might play a vital role in its drug resistant regulation. Consistently, several studies have suggested that miR-1246 might be involved in chemo-resistant regulation in cancers 35, 36.

To verify our hypothesis and make clear the relationship between miR-1246 and chemo-resistance in leukemia cells, some* in vitro* functional assays were performed. Our results showed that loss of miR-1246 could inhibit the survival and proliferation rate and enhance their chemo-sensitivity to ADM (Figure 2B). Both morphological characteristic and flow cytometry assay indicated that inhibition of miR-1246 could weaken the ability of chemo-resistant cells to resist drug invasion and lead them more sensitive to drug (Figure 2C and D). Consistent with this, overexpression of miR-1246 could develop chemo-resistant ability in drug-sensitive leukemia cells (Figure 3). In accordance with our results, many reports have confirmed the relationship between miR-1246 and apoptosis. Down-regulation of miR-1246 could inhibit tumor growth and promote apoptosis of cervical cancer cells 37. Overexpression of miR-1246 promoted UVB-induced apoptosis in HaCaT cells 38. Previous results indicated that lack of miR-1246 arrested the cell cycle at the G0/G1 phase 32. Similar to that, our cell cycle distribution assay proved that loss of miR-1246 could halt cells at the G0/G1 phase in chemo-resistant leukemia cells (Figure 2E). The *in vivo* results showed that the loss of miR-1246 could significantly decrease the cell proliferation ability and drug resistant ability of chemo-resistant leukemia cells (Figure 4). Based on these, we concluded that miR-1246 was overexpressed in chemo-resistant leukemia cells and contributed to enhance their drug resistance. Loss of miR-1246 could destroy their chemo-resistant ability by inhibiting proliferation, inducing apoptosis and disturbing cell cycle distribution.

In order to reveal the specific mechanism regulating drug resistance, bioinformatics target prediction tools were used to find the possible targets of miR-1246. According to the prediction results, one fragment at the 3'UTR of AXIN2 (313-320) and one fragment at the 3'UTR of GSK-3β (510-516) had putative seed-matching sites with miR-1246 (Figure 5E). In addition, our study also found that the loss of miR-1246 could up-regulate the expression level of AXIN2 and GSK-3β. Accordingly, overexpression of miR-1246 would inhibit their expression (Figure 5C and D). These results indicated that miR-1246 might regulate the drug sensitivity by targeting AXIN2 and GSK-3β. To confirm, a dual luciferase reporter assay was performed to validate whether AXIN2 and GSK-3β were direct targets of miR-1246. As shown in Figure 5F, compared with miRNA negative control and anti-sense orientation of fragments transfection, luciferase activity was significantly decreased in the cells transfected with miR-1246 and 3'UTR fragments in the sense orientation, which suggested that miR-1246 inhibited the expression of AXIN2 and GSK-3β by directly binding to the 3'UTR seed-matching sites. Multiple reports support our results. It has been known that miR-1246 could regulate the properties of cancer stemness in liver cancer through directly regulating AXIN2 and GSK-3β 39. miR-1246 could promote metastasis and invasion of A549 cells by targeting GSK-3β-mediated Wnt/β-catenin pathway 40. But, no study has provided the direct evidence of AXIN2 or GSK-3β in the regulation of miR-1246 in cell apoptosis or chemo-resistance. AXIN2 and GSK-3β are two key regulators of Wnt/β-catenin pathway. Wnt signals regulate self-renewal, metabolism, survival, proliferation and epithelial-to-mesenchymal transition (EMT), drug resistance of target cells 41. So, we inferred that miR-1246 influenced Wnt/β-catenin pathway by directly targeting AXIN2 and GSK-3β, and then played its drug resistant regulatory function. Our results confirmed that repression of miR-1246 also influenced the expression levels of major components of Wnt/β-catenin pathway and its downstream factors. Loss of miR-1246 deactivated Wnt2, β-catenin and c-Myc, while activated APC (Figure 5G). Based on this, we draw the following conclusions, miR-1246 influences chemo-sensitivity of leukemia cells via Wnt/β-catenin pathway by directly targeting AXIN2 and GSK-3β. Besides, repression of miR-1246 would also down-regulate the level of P-gp (Figure 5G). Another experiment also confirmed this result. Inhibition of miR-1246 could reduce ADM efflux ability in chemo-resistant leukemia cells. In accordance with our results, previous reports have showed that down-regulation of Wnt/β-catenin pathway could inhibit MDR1/P-gp expression by directly binding with MDR1 promoter and regulating its transcription 26. So, the present work prospectively proved that miR-1246 could regulate chemo-resistant ability of leukemia cells via Wnt/β-catenin pathway by directly targeting AXIN2, GSK-3β and indirectly regulating P-gp expression. Several studies have shown that miRNAs are attractive and potential therapeutic targets for tumor superior over other therapies (e.g., single proteins or small molecules) as a miRNA can potentially regulate complex biological processes 42. While miRNA inhibitors are negatively charged and do not readily bind to plasma membranes and enter the cell. Several chemical modifications (cholesterol, LNA) on antimiR have been found to improve cellular uptake and stability 42. Now, much progress has been made on the strategies for delivery miRNAs to their target tissues, include local delivery and systemic delivery (viral vectors, lipid vectors, nanoparticles, etc.) 43. Still, large amount of research occurring in this field will continue to improve development leading to more miRNA clinical trials.

Taken together, our findings provided a novel mechanistic therapeutic approach and demonstrated that miR-1246 affected cancer chemo-resistant ability in leukemia cells by directly targeting AXIN2 and GSK-3β and indirectly influencing the activity of Wnt/β-catenin pathway which could mediate the P-gp expression regulation. At the same time, miR-1246 could also generate chemo-resistant ability by enhancing cell proliferation ability and inhibiting apoptosis. Reports have proved that high miR-1246 expression is associated with poor prognosis in OSCC 35. Higher miR-1246 expression in lung cancer patients bearing tumors have shorter survival periods 36. Consequently, miR-1246 is a potential gene therapy target for the treatment of chemo-resistance in leukemia cells. Targeting miR-1246 may provide a novel diagnostic marker and an alternative approach for the drug resistance intervention in the treatment of leukemia.

## Figures and Tables

**Table 1:** Differentially expressed miRNA profile in K562/ADM cells compared to their parental cells.

K562/ADM/K562 10-fold up-regulation		K562/ADM/K562 0.2-fold down-regulation	
hsa-miR-1246	34.360681	hsa-miR-223	0.197751
hsa-miR-1973	29.754322	hsa-miR-101	0.1975354
hsa-miR-326	18.891941	hsa-miR-144	0.1892377
hsa-miR-4279	15.152674	hsa-miR-15a	0.1241903
hsv2-miR-H7-3p	13.703368	hsa-miR-3613-3p	0.1171199
sv40-miR-S1-5p	12.912907	hsa-miR-30c	0.1021111
hsa-miR-498	11.165278	hsa-miR-26b	0.0520349
hsa-miR-299-5p	10.954802	hsa-miR-142-5p	0.0349762
hsa-miR-1290	10.887637	hsa-miR-140-3p	0.0301875
hsa-miR-483-3p	10.408051	hsa-miR-29a	0.0138537
		hsa-miR-96	0.0138343
